# Toxic epidermal necrolysis complicated with respiratory failure in children: A case report

**DOI:** 10.1016/j.heliyon.2024.e25830

**Published:** 2024-02-05

**Authors:** Xiaoqian Chen, Suhua Jiang

**Affiliations:** Department of Pediatrics, The First People's Hospital of Foshan 528000, Guangdong. China

**Keywords:** Toxic epidermal necrolysis, respiratory failure, children, Case report

## Abstract

Stevens-Johnson syndrome or toxic epidermal necrolysis (TEN) is a severe skin and mucosal reaction that develops rapidly and has a high mortality rate. Its early identification and proper treatment are crucial to lowering the risk of death. Severe TEN can also lead to acute respiratory failure. This study probed the effect of early treatment on chronic airway damage in children with TEN complicated by respiratory failure. Three children diagnosed with TEN complicated by respiratory failure received interventions including high-dose glucocorticoids, gamma-globulin pulse therapy, and plasma exchange. One patient experienced recurrent lung infections, developed secondary chronic obstructive pulmonary disease, and eventually succumbed to respiratory failure despite skin improvement. The other two patients showed improvement after receiving combination treatment with a tumor necrosis factor-α (TNF-α) inhibitor. However, they also had concurrent chronic airway disease during the follow-up period. The exact mechanism underlying TEN remains uncertain. Children with TEN complicated by respiratory failure continue to experience chronic airway damage even after standard treatment. In future, multi-center clinical studies are warranted to investigate the impact of TNF-α inhibitors in children with TEN. Assessing the effectiveness and safety of targeted medications for TEN will provide more evidence regarding the prognosis of this disease.

## 1 Introduction

Stevens-Johnson syndrome (SJS) or toxic epidermal necrolysis (TEN) is a severe skin and mucosal reaction primarily triggered by medications. SJS/TEN is characterized by blistering and widespread skin loss, often involving multiple organs. Presently, SJS and TEN are regarded as the same condition with varying degrees of severity categorized on the basis of the extent of skin detachment relative to the body surface area (BSA): SJS (<10% BSA), SJS/TEN overlap (10–30% BSA), and TEN (>30% BSA) [1]. Mortality rates were reported to be 5.4% in SJS, 14.4% in SJS/TEN, and 15.3% in TEN [2]. The annual incidence rate of SJS and TEN is approximately 0.94–9.2 cases per million. Nonetheless, due to the rarity of this ailment and the challenges involved in conducting prospective studies in pediatric cohorts, particularly those displaying early respiratory tract damage and chronic lung disease, only a few TEN studies have been published. Epidemiological data on TEN complicated with respiratory failure are lacking in the literature. The sole report by Schmartz et al. addresses the respiratory complications of TEN in children [3]. The initial phase of the disease is characterized by flu-like symptoms such as fever and general malaise, followed by skin and mucous membrane pain, involving areas like the eyes, mouth, genitals, along with other systemic symptoms leading to skin detachment exceeding 30% of the BSA. Noteworthy mucosal injury in TEN encompasses the lips, mouth, conjunctiva, genital and anal mucosa, stomach, esophagus, and the bronchial system, and may lead to acute respiratory failure in severe instances. Instances involving necrosis of the airway mucocutaneous region demand special attention or endotracheal intubation, while a majority of TEN cases exhibit damage to multiple organs [4]. This study documents the clinical details of three patients with TEN complicated by respiratory failure in the Pediatric Intensive Care Unit of Foshan First People's Hospital, China during the period spanning 2017 to 2021. The patient characteristics are presented in Table 1. The cases are meticulously described and discussed in the context of pertinent domestic and international research.

**Table 1:** Characteristics of the patients included in the study.

Patient	Age(y)	Sex	Trigger	BSA (%)	SCORTEN [5]	Respiratory support	Blood test results	Main Treatments	Death
1	6	M	Aminophenazone	80	3 （HR: 153 beats/minGLU: 6.67 mg/dL, AB: 19.2 mmol/L, BUN: 3.73 mmol/L）	MV（D12）	WBC: 24.96 × 10^9/L (NEUT 84.60%), CRP: 63.8 mg/L, PCT: 116.48 ng/mL,	High-dose glucocorticosteroids Immunoglobulin infusion plasma exchange Meropenem and Vancomycin	yes
2	9	M	Lysine Acetylsalicylate	40	3 （HR: 141 beats/min GLU: 7.4 mg/dL, AB: 21.3 mmol/L, BUN: 3.33 mmol/L.）	MV（D5）	WBC: 21.79 × 10^9/L (NEUT: 88%), PCT: 3.34 ng/mL	High-dose glucocorticosteroids Immunoglobulin infusion Subcutaneous injections of TNF-α antagonist plasma exchange Ceftriaxone	no
3	7	F	Ibuprofen/Chinese medicine	60	4 （HR: 180 beats/min GLU: 13.2 mg/dL, AB: 19.9 mmol/L, BUN: 3.86 mmol/L）	MV（D3）	WBC: 13.72 × 10^9/L (NEUT: 88.7%), CRP: 58.39 mg/L, PCT: 46.79 ng/mL,	High-dose glucocorticosteroids Immunoglobulin infusion Subcutaneous injections of TNF-α antagonist plasma exchange Linezolid and Meropenem	no

NOTE: MV = mechanical ventilation, BSA = body surface area, WBC = white blood cell count, NEUT = neutrophils, PCT = procalcitonin, GLU = blood glucose, AB = actual bicarbonate, BUN = blood urea nitrogen, CRP=C-reactive protein HR = heart rate.

## 2 Case presentation

### 2.1 Patient 1

**Fig. 1:**
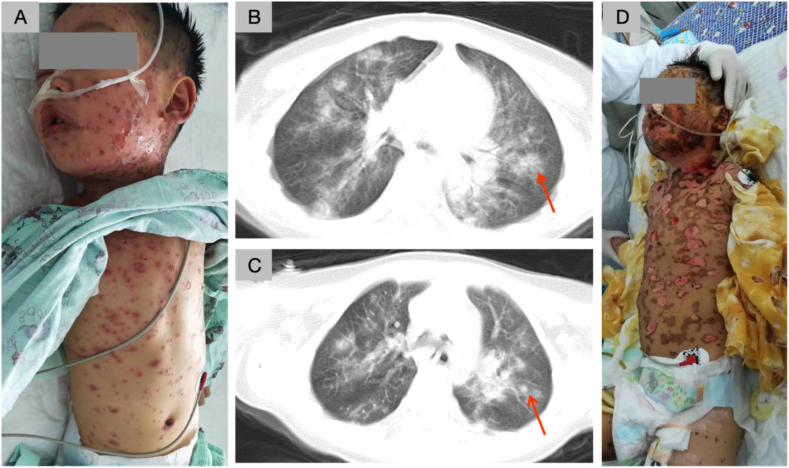
**Image of patient 1 at the time of first diagnosis of TEN after admission.** (A) Translucent blisters or ecchymosis-like changes appeared in the center of some rashes, especially on the head, face, trunk, and proximal limbs. (B–C) Lung computed tomography (CT; Philips Ingenuity microplate CT) was performed after endotracheal intubation, and multiple patchy high-density shadows, bronchial thickening, and local lung consolidation were observed in both lungs. Due to the presence of jitter in the CT radiographs of the child, the image appears fuzzy, and the annotated image contains artifacts. The red arrow indicates local lung consolidation. (D) After treatment in the hospital, the skin and mucous membrane damage worsened. (For interpretation of the references to colour in this figure legend, the reader is referred to the Web version of this article.)

A 6-year-old boy was admitted to the local hospital on August 24, 2017, due to a complaint of "cough and fever for two days and rash for one day." His condition began with a high fever that started two days ago, reaching a peak temperature of 40 °C. In addition to the fever, he displayed a cough accompanied by yellow-green sputum. The patient was treated with high-dose sodium succinate (20 mg/kg.d), immunoglobulin shock (2 g/kg), and plasma exchange (5 times, 50–80 mL/kg). Following the administration of cefuroxime axetil through intravenous infusion and Antongdine via intramuscular injection, the patient developed a scattered red maculopapular rash across his entire body one day ago. The rash gradually spread to his head, face, trunk, oral mucosa, and conjunctiva. He received treatment at a local outpatient clinic, including intravenous injection of dexamethasone. However, after two days of receiving intravenous piperacillin, his symptoms did not show significant improvement, prompting his transfer to our hospital. Upon admission, a physical examination revealed a heart rate of 126 beats/min. The BSA detached was 80% (Fig. 1a). Some of the rashes exhibited translucent blisters or ecchymosis-like changes in their centers, especially on the head, face, trunk, and proximal limbs. Nissl's sign was positive, and there was conjunctival hyperemia with pale yellow secretions. Additionally, the patient had restricted mouth opening, swollen and chapped lips, erosion in the buccal and palate mucosa, ulcers, and a body surface covered with yellowish attachments. Gingival swelling was also observed. The patient was diagnosed with TEN. Despite treatment in the hospital, both skin and mucous membrane damage worsened (Fig. 1d), and the patient experienced high fever, shortness of breath, and cough. Arterial blood gas analysis indicated a pH of 7.33, PaCO_2_ of 42.3 mmHg, PaO_2_ of 55.6 mmHg, and HCO_3_ of 21.0 mmol/L, indicating hypoxemia and metabolic acidosis. Eventually, on the 10th day of admission, the patient suffered respiratory and cardiac arrest. After cardiopulmonary resuscitation (CPR), he exhibited an increased respiratory rate, chest retractions, and hypoxemia. As a result, the patient underwent tracheal intubation to facilitate ventilator-assisted breathing. Bronchoscopy revealed detached tracheal intima that could be extracted. Metagenomic testing of bronchoalveolar lavage fluid identified the presence of *Acinetobacter baumannii* and *Pseudomonas aeruginosa*. There was no positive result in sputum culture at admission. Therefore, a secondary infection occurring after immune injury was considered as the likely explanation for these microbiological test results. Computed tomography (CT) (Fig. 1b and c) indicated multiple patchy high-density shadows, bronchial thickening, and local lung consolidation in both lungs.

Following 76 hours of mechanical ventilator support, the patient exhibited improvement and subsequently underwent extubation. He was transferred to another medical facility for continued care. However, he encountered another episode of respiratory and cardiac arrest there, necessitating re-initiation of mechanical ventilation. After 35 days of hospitalization, he was discharged. Nonetheless, after a span of two months, he was readmitted due to respiratory symptoms including shortness of breath, cough, and respiratory distress. A culture of bronchoalveolar lavage fluid revealed the presence of *Candida albicans* and *Moraxella catarrhalis*. As time progressed, he developed corneal detachment in both eyes, resulting in blindness. Ultimately, his demise ensued due to severe pneumonia, chronic obstructive pulmonary disease, and respiratory failure.

### 2.2 Patient 2

**Fig. 2:**
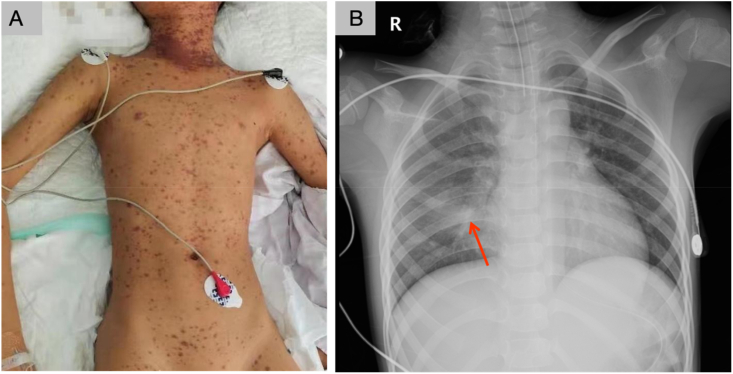
**Images for patient 2 diagnosed with TEN.** (A) The day after admission, a scattered red rash appeared on the neck that gradually spread to the face and body. (B) A chest X-ray taken the day after admission revealed local consolidation of the lower right lung. The red arrow indicates local lung consolidation. (For interpretation of the references to colour in this figure legend, the reader is referred to the Web version of this article.)

A 9-year-old boy was admitted to a hospital on February 20, 2021, owing to a history of "fever for three days, rash for two days, and worsening condition for one day." Patient 2 was treated with high-dose glucocorticoids (20 mg/kg.d), immunoglobulin shock (2 g/kg), subcutaneous injection of TNF-α antagonist (12.5 mg/day) on the first and second day, and plasma exchange (50–80 mL/kg for 5 days). On the fourth day of admission, the patient experienced high fever and received treatment in the form of oral cefuroxime sodium, as well as intramuscular injections of andrographolide and lysine aetylsalicylate. The following day, he presented with conjunctival hyperemia, sore throat, and a few small blisters at the corner of his mouth, accompanied by lip swelling. A yellow discharge emerged from his eyes. A red rash, initially appearing on his neck, gradually extended to his face and body (Fig. 2a). Concurrently, he experienced a mild cough with phlegm. Since the condition did not improve with treatment involving intravenous infusion of cefuroxime sodium, mezlocillin sodium, sulbactam sodium, and acyclovir, he was transferred to our hospital. The patient had a vesicular rash on both cheeks and numerous brown purpura-like maculopapular eruptions on the ears, jaw, neck, trunk, back, and extremities. These rashes exhibited tenderness, did not release exudate, remained unaffected under pressure, and exhibited a positive Nissl's sign. The patient's eyelids were erythematous and conjunctival hyperemia was evident. The palpebral conjunctiva featured a pseudomembrane, and the cornea displayed a rough texture. His lips were chapped, and substantial purple-black scabs were visible on the upper and lower lips. The pharynx exhibited congestion. A rash appeared on the penis, accompanied by epidermal peeling, a swollen and red urethra, and visible white secretions.

On the second day post-admission, he developed a cough, shortness of breath, chest retractions, and hypoxemia. A chest radiograph (Fig. 2b) revealed local consolidation in the right lower lung. Arterial blood gas analysis showed a pH of 7.402, PaCO_2_ of 47.9 mmHg, PaO_2_ of 59.4 mmHg and HCO_3_ of 29.7 mmol/L, indicating hypoxemia and metabolic alkalosis. Tracheal intubation was undertaken for 142 hours with ventilator support. On the 11th day of treatment, the rash displayed no worsening, and the vital signs of the patient remained stable. After a span of 24 days, he was discharged from the hospital. A one-year follow-up revealed no exercise intolerance and the absence of lung rales, but his pulmonary function showed a mild early-stage obstructive ventilation disturbance. After administration of glucocorticoids (via inhalation) for 6 months, his pulmonary function became normal.

### 2.3 Patient 3

**Fig. 3:**
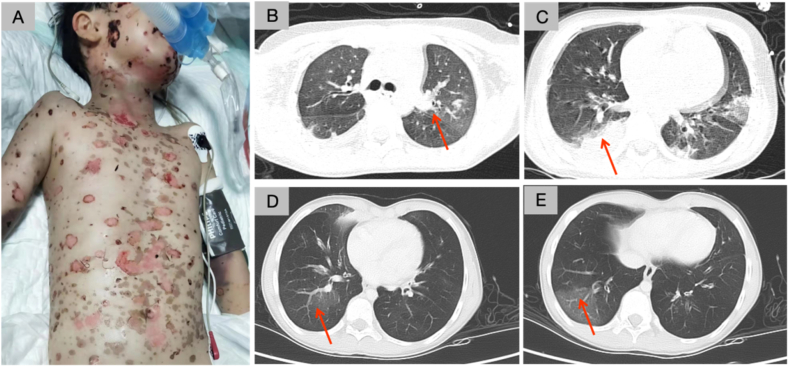
**Images for patient 3 diagnosed with TEN.** (A) Scattered dark red herpes and maculopapular rash all over the body. (B–C) Copmputed tomography (CT) examination after endotracheal intubation on ventilator showed multiple flaky solid shadows in both lungs (as shown by the red arrow). (D–E) Reexamination of CT results one year after discharge showed multiple patchy blurred ground glass density shadows in both lungs (as shown by the red arrow). (For interpretation of the references to colour in this figure legend, the reader is referred to the Web version of this article.)

A 7-year-old girl was admitted to the hospital on September 30, 2021, due to a reported history of "fever with rash for two days." The patient experienced an abrupt onset of high fever two days prior, accompanied by scattered herpes and maculopapular rash. Additional symptoms included cough, expectoration, intermittent vomiting, and forehead pain. Patient 3 was treated with a high-dose hormone shock (20 mg/kg.d), gamma globulin shock (2 g/kg), subcutaneous injection of TNF-α antagonist (20 mg/day) on the first and second day, and plasma exchange (50–80 mL/kg for 5 days). Treatment involved the use of oral ibuprofen, ribavirin tablets, amoxicillin and clavulanate potassium, mezlocillin through intravenous drip, and traditional Chinese medicine. Upon admission, a physical examination disclosed the presence of dark red scattered herpes and maculopapular rash across her entire body (Fig. 3a). There was bilateral conjunctival hyperemia with purulent secretion and swollen and painful dark red lips. Multiple herpetic lesions and ulcers were detected in the pharyngeal isthmus and oral mucosa. Furthermore, the patient displayed shortness of breath and moist rales in both lungs. The patient was diagnosed with TEN. In conjunction with hormone, immunoglobulin, and tumor necrosis factor (TNF-α) antagonist treatment, the patient underwent therapy involving tobramycin and dexamethasone eye drops, hydrogen peroxide mouthwash, diclofenac sodium spray for inflammation reduction and pain relief, and the application of an ointment containing boric acid, zinc oxide, and the monoterpene borneol to the blisters. On the second day of hospitalization, the patient displayed respiratory distress, shortness of breath, and nasal flaring. Arterial blood gas analysis revealed a pH of 7.357, PaCO_2_ of 32.0 mmHg, PaO_2_ of 54.9 mmHg, standard base excess −7.6 mmol/L and HCO_3_ 17.9 mmol/L, consistent with hypoxemia, metabolic acidosis and respiratory alkalosis. Bronchoscopy revealed a substantial quantity of white viscous secretion, leading to tracheal intubation and ventilator support. Lung CT scans (Fig. 3b and c) depicted multiple lamellar solid shadows in both lungs. Ventilator assistance was ceased on the 15th day after witnessing improvement. However, on the 17th day of hospitalization, shortness of breath exacerbated, necessitating a reintroduction of tracheal intubation and ventilator support. After 599 hours, the ventilator was withdrawn. On the 40th day of treatment, the rash exhibited marked regression, while the vital signs of the patient remained stable. Subsequently, after a span of 79 days, the patient was discharged. Throughout the one-year follow-up, the patient faced recurring cough, shortness of breath, and wheezing. She required rest after walking 20–30 m, yet these symptoms were mitigated through oral medication and nebulization. The patient could not complete the forced pulmonary function test. Re-examination of CT scans (Fig. 3d and e) highlighted the presence of multiple patchy, fuzzy, ground glass density shadows in both lungs.

## 3 Discussion and conclusions

TEN is an infrequent and severe blistering reaction to drugs, initially described by Alan Lyell in 1956. Prior investigations, both domestically and internationally, have focused more on the aftermath of TEN in adults, and have focused on the genital mucosa and ocular conditions [5]. Of note, penile or vaginal fusion with severe functional compromise has been a potentially serious complication of TEN. However, respiratory symptoms are also a cause for concern, especially in children. Amongst the three cases examined in this study, respiratory failure was diagnosed in all patients during the course of the disease, necessitating ventilator-assisted breathing. Nonetheless, medication regimens differed slightly among the cases, subsequently yielding diverse outcomes. The risk factors for TEN include immune dysregulation, concurrent malignancy, and genetic susceptibility. Drugs are the primary trigger for SJS/TEN, particularly antimicrobials, anti-epileptics, allopurinol, and non-steroidal anti-inflammatory drugs (NSAIDs). A recent analysis [6] of adverse effects (AEs) revealed that 24.2% of these incidents occurred in children. Moreover, 15.5% of all cases with fatal outcomes were attributed to SJS/TEN, while 6.2% of AEs consisted of prodromes and symptoms indicative of potentially life-threatening SJS/TEN. These statistics underline the elevated occurrence of SJS/TEN in children. In the current study, all three children had a history of NSAID usage before the onset of the rash, hinting at NSAIDs as a common trigger for SJS/TEN in children. Ma et al. reported that NSAIDs (or so-called "cold medicine") were the most causative drugs for SJS/TEN patients with severe ocular complications. In addition, the HLA-A02:07 and HLA-B46:01 alleles were significantly associated with Han Chinese patients with NSAID-induced SJS/TEN. These findings are distinct from those of previous reports on other ethnic groups, demonstrating that genetic diversity exists in SJS/TEN pathogenesis among different ethnic groups. This diversity may regulate the outcome of the disease and be a potential predictive diagnostic tool for prognosis [7]. While antibiotics were administered to all three children during the early stages of treatment, the previous use of similar drugs had not caused skin symptoms. It is worth investigating whether Chinese herbal medicines, simultaneously administered in the case of patient 3, may potentially induce TEN.

Primary treatments for TEN include glucocorticoids, immunoglobulins, immunosuppressive agents, tumor necrosis factor-α (TNF-α) antagonists, and plasma exchange [8]. Although we could locate studies on the current status of TNF antagonists in the treatment of connective tissue disease-related interstitial lung disease, we were unable to find any reports describing a role of TNF inhibitors in causing interstitial lung disease. There is also no causal relationship between TNFα inhibitors and AEs reported in the literature [9]. The utilization of early glucocorticoid pulse therapy remains a topic of debate due to concerns regarding increased bacterial infections and delayed skin re-epithelialization associated with glucocorticoid use. Nonetheless, several experts assert that the application of glucocorticoids has substantially curbed disease progression. Early glucocorticoid pulse therapy does not alter the stabilization period of the disease, or the time required for re-epithelialization, and does not lead to heightened sepsis rates [10]. Extensive doses of glucocorticoids may suppress lymphocytes, and infections may arise due to skin and mucous membrane impairment. Consequently, the use of immune-modulating drugs may be necessary to mitigate the risk of infection during glucocorticoid treatment.

Intravenous immunoglobulin (IVIG) therapy has demonstrated notable benefits in the treatment of SJS/TEN by impeding keratinocyte apoptosis [11]. The combination of glucocorticoids with IVIG therapy has shown the potential to lower mortality rates. Reinforced supportive care can mitigate skin infections in the denuded region, thereby effectively decreasing the incidence of complications and improving prognosis. Increasing evidence underscores the advantages of TNF-α antagonists in managing SJS/TEN. A randomized controlled trial conducted by Wang et al. in Taiwan [12] showcased improved clinical outcomes through the use of TNF-α antagonist (etanercept) therapy for SJS/TEN, that included complications such as mortality, skin healing duration, and gastrointestinal bleeding. The median healing time for TEN with TNF-α antagonist treatment ranged from 8 to 9 days, necessitating prompt initiation of treatment [13]. Among the cases discussed here, the patient who eventually succumbed to late complications did not receive TNF-α antagonist combination therapy. This implies that TNF-α antagonists positively influence prognosis but must be administered in a timely manner. Significantly protracted mechanical ventilation was observed in all three cases. TEN can impact the oropharyngeal and bronchial mucosa, leading to the occurrence of residual epithelial fragments, difficulties in swallowing and expelling secretions, atelectasis development, and eventual acute respiratory failure. This situation may necessitate mechanical ventilation. However, extended periods of mechanical ventilation may result in complications, including the potential for oxygen to exacerbate pulmonary fibrosis. As a countermeasure, antioxidant therapy may be employed in children who require prolonged mechanical ventilation to diminish the risk of chronic lung disease.

Patient 2 had no symptoms of chronic respiratory failure, and lung function improved after treatment, so the presence of bronchiolitis obliterans (BO) was not considered. Patient 3 exhibited evident airway sequelae during the prolonged follow-up, with CT scans revealing ground-glass density shadows, indicative of BO. Respiratory epithelial detachment and recurrent respiratory tract infection are the main reasons for BO. The underlying mechanism for BO secondary to SJS/TEN remains obscure; we speculate that it may be attributed to immune complex deposition damaging bronchial epithelial cells and mucosa. Aberrant immune responses coupled with respiratory infections may play a pivotal role in BO development in SJS/TEN [14]. A more recent report from the same group has revealed that systemic etanercept promoted skin and corneal wound healing in patients with SJS/TEN having cancer who survived the acute episode. However, there are windows of treatment opportunity, beyond which the benefit of TNF-α antagonist treatment is greatly reduced [15]. BO may manifest in patients with TEN at varying stages, thereby underscoring the importance of prolonged monitoring for persistent respiratory complications, even after TEN recovery. The majority of TEN cases stem from drug-related adverse reactions, characterized by lesions on the face, extremities, genitals, trunk, oral mucosa, and eyes. Patients necessitating mechanical ventilation exhibit a substantial in-hospital mortality rate of 57%. Consequently, patients with acute respiratory failure should be promptly identified and referred to the ICU for specialized intervention [16]. The precise pathological mechanism underlying TEN remains elusive, and children with respiratory failure have a higher likelihood of developing chronic airway lesions despite conventional treatment. A study by Schmartz et al. [17] observed enduring sequelae in 95% of the 22 children diagnosed with TEN.

Etanercept promotes skin and corneal wound healing in surviving patients with SJS/TEN having cancer after an acute attack. However, there is a therapeutic opportunity window for TNF-α antagonist treatment. Beyond this therapeutic opportunity window, the benefits of TNF-α antagonist treatment are greatly reduced. Therefore, once TEN is considered, TNF-α antagonists should be initiated as soon as possible. Currently, only a few cases of respiratory failure caused by TEN have been described in the literature. There is a pressing need for multi-center clinical studies to explore the efficacy and safety of TNF-α antagonists in children affected by TEN, thereby contributing more comprehensive evidence for prognosis after TEN targeted drug therapy.

## Ethics approval

The research related to human use has been complied with all the relevant national regulations, institutional policies and in accordance with the tenets of the Helsinki Declaration, and has been approved by the authors’ institutional review board or equivalent committee.

## Informed consent

Informed consent was acquired in the manuscript file from the guardian AND that guardians consented to the publishing of all images, clinical data, and other data included in the manuscript.

## Funding

This study received funding from the Foshan 14th Five-Year High-Level Key Specialized Subject Construction.

## Data Availability Statements

Data sharing is not applicable to this article as no datasets were generated or analyzed during the current study.

## CRediT authorship contribution statement

**Xiaoqian Chen:** Writing – original draft, Investigation, Formal analysis, Data curation. **Suhua Jiang:** Writing – review & editing, Supervision, Conceptualization.

## Declaration of competing interest

The authors declare that they have no known competing financial interests or personal relationships that could have appeared to influence the work reported in this paper.

## List of abbreviations

Stevens-Johnson syndrome: SJS
Toxic epidermal necrolysis: TEN
Body surface area: BSA
Non-steroidal anti-inflammatory drugs: NSAIDs
Intra-Venous Immunoglobulin: IVIG
Bronchiolitis obliterans: BO

## References

[1] White K.D. (2018). SJS/TEN 2017: Building Multidisciplinary Networks to drive Science and Translation. J Allergy Clin Immunol Pract.

[2] Hsu D.Y. (2016). Morbidity and mortality of Stevens-Johnson syndrome and toxic epidermal necrolysis in United States adults. J Invest Dermatol.

[3] Schmartz S. (2023). Respiratory complications in pediatric epidermal necrolysis: a retrospective study of 22 cases. J Am Acad Dermatol.

[4] Wetter D.A., Camilleri M.J. (2010). Clinical, Etiologic, and Histopathologic Features of Stevens-Johnson syndrome during an 8-year period at Mayo clinic. Mayo Clinic Proceedings.

[5] Yang Y. (2009). Combination therapy of intravenous immunoglobulin and corticosteroid in the treatment of toxic epidermal necrolysis and Stevens-Johnson syndrome: a retrospective comparative study in China. Int J Dermatol.

[6] Zavala (2018). How does SCORTEN Score?. Journal of burn care & research: official publication of the American Burn Association.

[7] Ma K.S. (2022). Human leucocyte antigen association of patients with Stevens-Johnson syndrome/toxic epidermal necrolysis with severe ocular complications in Han Chinese. Br J Ophthalmol.

[8] Lee Walsh, Creamer (2017). Long term complications of Stevens-Johnson syndrome/Toxic epidermal necrolysis: the spectrum of chronic problems in patients who survive an episode of SJS/TEN necessitates multi-disciplinary follow up. Brit J Dermatol.

[9] Li M. (2023). Characteristic analysis of adverse reactions of five anti-TNFa agents: a descriptive analysis from WHO-VigiAccess. Front Pharmacol.

[10] Owen C.E., Jones J.M. (2021). Recognition and Management of severe cutaneous adverse drug reactions (including drug reaction with Eosinophilia and systemic symptoms, Stevens-Johnson syndrome, and toxic epidermal necrolysis). Med Clin North Am.

[11] Popiolek I., Piotrowicz-Wojcik K., Porebski G. (2019). Hypersensitivity reactions in serious adverse Events reported for Paracetamol in the EudraVigilance Database, 2007(-)2018. Pharmacy (Basel).

[12] Estrella-Alonso A. (2017). Toxic epidermal necrolysis: a paradigm of critical illness. Rev Bras Ter Intensiva.

[13] Emre S. (2019). Intravenous immunoglobulin treatment: where do dermatologists stand?. Dermatologic Therapy.

[14] Wang C.W. (2018). Randomized, controlled trial of TNF-alpha antagonist in CTL-mediated severe cutaneous adverse reactions. J Clin Invest.

[15] Ma K.S. (2021). Ocular manifestations of anti-neoplastic immune checkpoint inhibitor-associated Stevens-Johnson syndrome/toxic epidermal necrolysis in cancer patients. Ocul Surf.

[16] Keerty D. (2020). Immune-Mediated toxic epidermal necrolysis. Cureus.

[17] Zhang R. (2015). Mechanical Stress and the Induction of lung Fibrosis via the Midkine Signaling Pathway. Am J Respir Crit Care Med.

